# LRP5 regulates the expression of STK40, a new potential target in triple-negative breast cancers

**DOI:** 10.18632/oncotarget.25187

**Published:** 2018-04-27

**Authors:** Sylvie Maubant, Tania Tahtouh, Amélie Brisson, Virginie Maire, Fariba Némati, Bruno Tesson, Mengliang Ye, Guillem Rigaill, Maïté Noizet, Aurélie Dumont, David Gentien, Bérengère Marty-Prouvost, Leanne de Koning, Sardar Faisal Mahmood, Didier Decaudin, Francisco Cruzalegui, Gordon C. Tucker, Sergio Roman-Roman, Thierry Dubois

**Affiliations:** ^1^ Institut Curie, PSL Research University, Translational Research Department, Breast Cancer Biology Group, Paris, France; ^2^ Institut Curie, PSL Research University, Translational Research Department, Preclinical Investigation Laboratory, Paris, France; ^3^ Institut Curie, PSL Research University, INSERM U900, Paris, France; ^4^ Institute of Plant Sciences Paris-Saclay (IPS2), UMR 9213/UMR 1403, CNRS, INRA, Université Paris-Sud, Université d’Evry, Université Paris-Diderot, Sorbonne Paris-Cité, Orsay, France; ^5^ Laboratoire de Mathématiques et Modélisation d’Evry (LaMME), Université d’Evry Val d’Essonne, UMR CNRS 8071, ENSIIE, USC INRA, Évry, France; ^6^ Institut Curie, PSL Research University, Translational Research Department, Genomics Platform, Paris, France; ^7^ Institut Curie, PSL Research University, Translational Research Department, Reverse-Phase Protein Array Platform, Paris, France; ^8^ Oncology Research and Development Unit, Institut de Recherches SERVIER, Croissy-Sur-Seine, France; ^9^ Institut Curie, PSL Research University, Translational Research Department, Paris, France

**Keywords:** triple-negative breast cancer, LRP5, LRP6, STK40, targeted therapy

## Abstract

Triple-negative breast cancers (TNBCs) account for a large proportion of breast cancer deaths, due to the high rate of recurrence from residual, resistant tumor cells. New treatments are needed, to bypass chemoresistance and improve survival. The WNT pathway, which is activated in TNBCs, has been identified as an attractive pathway for treatment targeting. We analyzed expression of the WNT coreceptors LRP5 and LRP6 in human breast cancer samples. As previously described, LRP6 was overexpressed in TNBCs. However, we also showed, for the first time, that LRP5 was overexpressed in TNBCs too. The knockdown of LRP5 or LRP6 decreased tumorigenesis *in vitro* and *in vivo*, identifying both receptors as potential treatment targets in TNBC. The apoptotic effect of LRP5 knockdown was more robust than that of LRP6 depletion. We analyzed and compared the transcriptomes of cells depleted of LRP5 or LRP6, to identify genes specifically deregulated by LRP5 potentially implicated in cell death. We identified serine/threonine kinase 40 (STK40) as one of two genes specifically downregulated soon after LRP5 depletion. STK40 was found to be overexpressed in TNBCs, relative to other breast cancer subtypes, and in various other tumor types. STK40 depletion decreased cell viability and colony formation, and induced the apoptosis of TNBC cells. In addition, STK40 knockdown impaired growth in an anchorage-independent manner *in vitro* and slowed tumor growth *in vivo*. These findings identify the largely uncharacterized putative protein kinase STK40 as a novel candidate treatment target for TNBC.

## INTRODUCTION

Breast cancer is the most common type of cancer in women. It is a heterogeneous disease, with four main subtypes defined on the basis of gene expression profiles: luminal A, luminal B, human epidermal growth factor receptor 2 (HER2)-overexpressing and basal-like [1, 2]. The basal-like subgroup is itself very heterogeneous, with at least six distinct subtypes [3–6]. Basal-like tumors resemble triple-negative breast cancers (TNBCs), which pathologists identify on the basis of their absence of expression of estrogen (ER) and progesterone receptors and lack of HER2 overexpression [7, 8]. TNBC patients respond well to conventional chemotherapies, but this subtype nevertheless accounts for a large proportion of breast cancer deaths, due to high rates of recurrence from residual, resistant tumor cells [8]. New treatments are therefore required, to overcome chemoresistance and improve survival [4, 5, 7, 9–11].

The canonical Wnt signaling pathway plays an important role in embryonic development and tumorigenesis [12–17]. This pathway underlies the properties of breast (cancer) stem cells, and is involved in mammary gland development and breast tumorigenesis [18–23], particularly in TNBC [24–35]. Unlike colorectal cancers, breast cancers present no mutations of molecules involved in the WNT pathway, such as adenomatous polyposis coli (APC) or β-catenin. By contrast, some transmembrane receptors, such as Frizzled receptor 6 (Fzd6), Fzd7, and low-density lipoprotein receptor-related protein 6 (LRP6), may be overexpressed, leading to activation of the canonical Wnt signaling pathway in TNBC [26–28, 31, 36]. LRP6 is overexpressed in TNBC, favoring cell proliferation, migration, invasion and tumor growth [27, 37–39]. Antibodies targeting LRP6 [40–42] or Fzd7 [28, 43–45] have been reported to display anti-tumor activity *in vivo*. Overall, these results suggest that the canonical WNT pathway is an attractive target for the treatment of TNBC, possibly through the invalidation of overexpressed transmembrane receptors [33, 36].

We analyzed genomic, transcriptomic and proteomic data from our cohort of 154 breast cancer samples [46, 47]. We observed the overexpression, not only of LRP6, as previously described [26, 27], but also of LRP5 in TNBCs relative to other breast cancer subtypes. The LRP5 and LRP6 coreceptors are frequently considered as a single entity, LRP5/6. However, they may have different functions in certain contexts [48–51]. We therefore investigated the effects of the specific modulation of LRP5 or LRP6 expression on cell viability and tumorigenesis in TNBC cell lines. We found that LRP5 and LRP6 had similar tumorigenic properties, but that LRP5 depletion induced apoptosis more effectively than LRP6 invalidation in the cell lines analyzed. We investigated this phenomenon further, by analyzing and comparing the transcriptomes of cells depleted of LRP5 and cells depleted of LRP6. We found that serine/threonine kinase 40 (STK40) was specifically downregulated in conditions of LRP5 depletion. The depletion of STK40, like that of LRP5, induced apoptosis and decreased cell viability, colony formation and growth in an anchorage-independent manner. We found that the depletion of LRP5, LRP6 or STK40 slowed tumor growth in an MDA-MB-468-derived xenograft model. In conclusion, both LRP5 and LRP6, consistent with previous reports for LRP6 [26, 27], are good candidates for therapeutic intervention. Our results also identify the little-studied and poorly characterized putative protein kinase STK40 as an essential protein for cell survival and a previously unconsidered candidate target for the treatment of TNBC.

## RESULTS

### LRP5 and LRP6 are overexpressed in triple-negative breast cancers relative to other breast cancer subtypes

**Figure 1:**
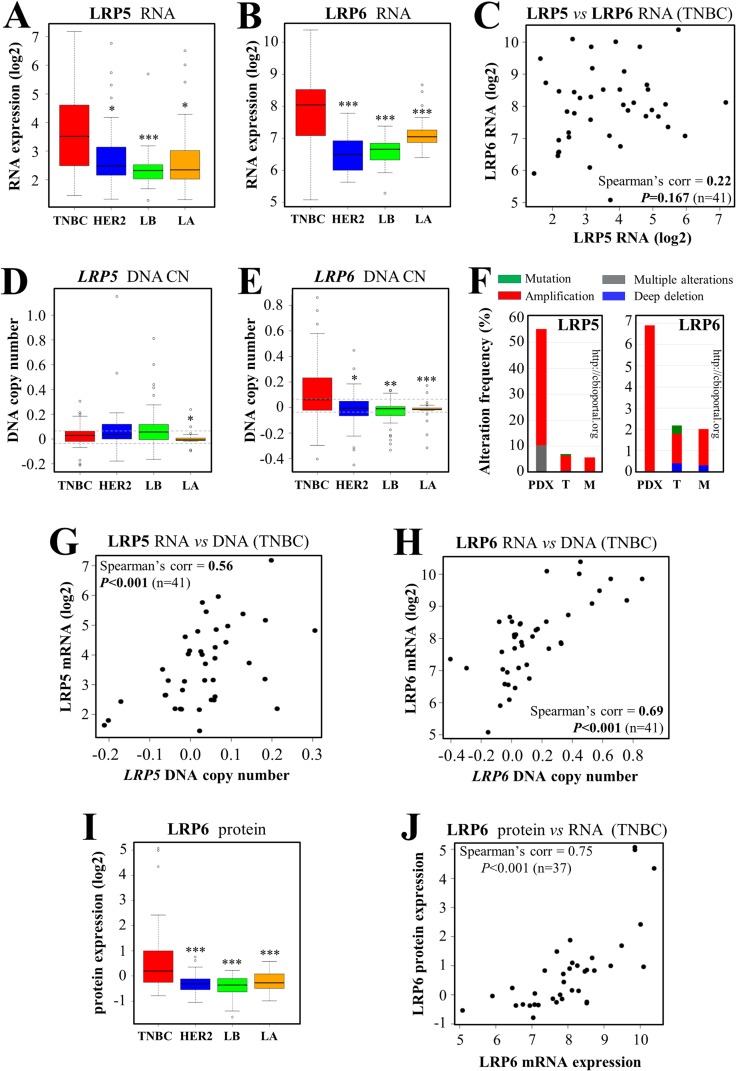
LRP5 and LRP6 are more strongly expressed in TNBC than in other breast cancer subtypes We assessed the expression of LRP5 and LRP6 in various subtypes of breast tumors from our cohort [46, 47]: TNBC, HER2^+^/ER^-^ (HER2), luminal B (LB) and luminal A (LA) tumors. **(A-B)** RNA microarray analysis was performed to assess the levels of LRP5 (A) and LRP6 (B) mRNA. **(C)** Correlation between the levels of LRP5 and LRP6 RNA in the TNBC subgroup. Each TNBC tumor from our cohort (*n*=41) is represented by a dot. **(D-E)** DNA copy number (CN) of the *LRP5* (D) and *LRP6* (E) genes. The smoothed segmented copy number signal is presented in boxplots, with dashed lines indicating the thresholds retained for the detection of DNA CN gains and losses. **(F)** We queried the cBio Cancer Genomics Portal (http://cbioportal.org) [54, 55], to determine whether LRP5 and LRP6 were altered in breast cancer. The graphs imported from cbioportal show the changes in frequency for LRP5 (left panel) and LRP6 (right panel) in 3 publicly available breast cancer cohorts: patient-derived xenograft models (PDX) [58], TCGA (T) [56] and METABRIC (M) [57]. Color code: green: mutation; gray: multiple alterations; blue: deletion; red: amplification. **(G)** Correlation between LRP5 RNA levels and *LRP5* DNA CN in TNBC. **(H)** Correlation between LRP6 RNA levels and *LRP6* DNA CN in TNBCs. **(I)** LRP6 protein levels were assessed with a reverse-phase protein array (RPPA). **(J)** Correlation between LRP6 RNA and protein levels within the TNBC subgroup. Each tumor (*n*=37) is represented by a dot. The values obtained for the relative quantification of protein and mRNA were log-transformed and are shown as box plots (A-B, D-E, I). Outliers are shown within each population studied (open circles) (A-B, D-E, I). ^*^P<0.05, ^**^P<0.01, ^***^P<0.001 (comparisons with TNBC: A-B, D-E, I).

LRP6 has been identified as a possible treatment target in TNBC [27, 36, 52, 53]. LRP6 and LRP5 are coreceptors. We, therefore, analyzed the expression of both LRP5 and LRP6 in our set of breast cancer biopsy specimens [46, 47]. Transcriptomic analysis revealed that both these receptors were expressed more strongly in TNBC than in other breast cancer subtypes (Figure 1A, 1B). We investigated the similarity of their expression in each TNBC sample (Figure 1C). There was a trend towards similar expression patterns, but the correlation between LRP5 and LRP6 RNA levels was not statistically significant (Figure 1C, *P*=0.167). In particular, some tumor samples had high levels of LRP6 RNA and low levels of LRP5 RNA (Figure 1C, top left corner), whereas the opposite pattern was observed for others (Figure 1C, bottom right corner). *LRP5* DNA copy number (CN) in TNBC was higher than that in luminal A tumors only (Figure 1D), but *LRP6* DNA CN was higher in TNBC than in the other breast cancer subtypes (Figure 1E). An exploration of the cBioPortal for cancer genomics (http://www.cbioportal.org) [54, 55] revealed that LRP5 was more frequently altered in breast cancers, mostly through amplification (TCGA [56] and METABRIC [57] cohorts), than LRP6 (Figure 1F). This phenomenon was particularly pronounced in breast cancer patient-derived xenograft (PDX) models [58], in which alterations of LRP5 were observed in more than 50% of cases, versus less than 7% of cases for LRP6 (Figure 1F). RNA levels and DNA CN were correlated in TNBC, for both LRP5 (Figure 1G) and LRP6 (Figure 1H) (*P*<0.001), this correlation being stronger for LRP6 (correlation coefficient=0.69) than for LRP5 (correlation coefficient=0.56). The overexpression of LRP6 in TNBC was confirmed at the protein level, by reverse-phase protein array (RPPA) analysis (Figure 1I). Unfortunately, it was not possible to analyze LRP5 protein levels, because none of the available antibodies suitable for western blotting was specific enough for RPPA analysis. We found a highly significant correlation between LRP6 protein and mRNA levels in TNBC (Figure 1J, correlation coefficient=0.75, *P*<0.001).

Together, these results indicate that both LRP5 and LRP6 are overexpressed in TNBC relative to other breast cancer subtypes.

### The silencing of LRP5 or LRP6 in breast cancer cells decreases cell viability and colony formation *in vitro*

**Figure 2:**
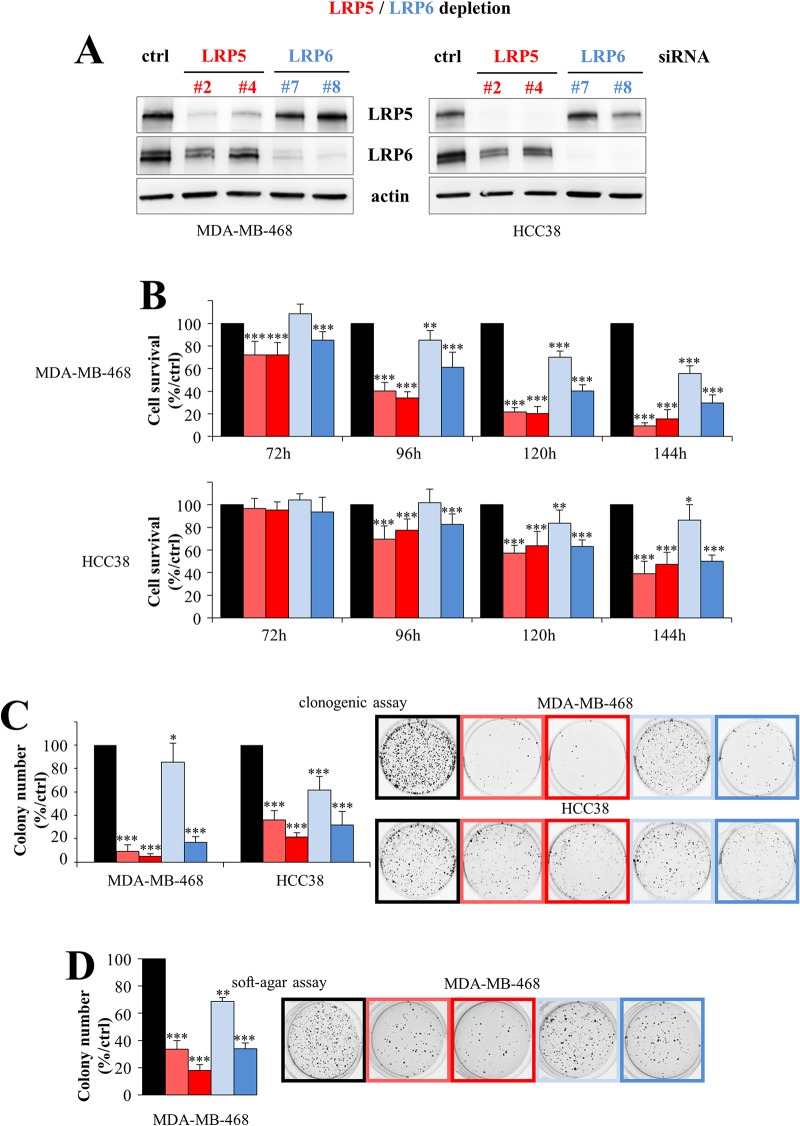
LRP5 and LRP6 are essential for the survival of breast cancer cells Two TNBC cell lines (MDA-MB-468, HCC38) with high levels of the LRP5 and LRP6 proteins were transfected with one of two different siRNAs against LRP5 (^#^2 and ^#^4, red), one of two siRNAs against LRP6 (^#^7 and ^#^8, blue) or with a control siRNA (black). **(A)** Levels of LRP5 or LRP6 protein were evaluated by western blotting 120 hours after transfection. Actin was used as a loading control. One experiment of the three performed, all of which gave similar results, is shown. **(B)** Cell viability was assessed in MTT (MDA-MB-468) or WST-1 (HCC38) assays, 72 to 144 h after transfection. Results are presented as percent cell viability relative to cells treated with control siRNA (100%). The data shown are means + SD from at least three independent experiments. **(C)** Cells were transfected and transferred to six-well plates, in which they were cultured for 6 to 10 days, until colony formation. The number of colonies is expressed as a percentage relative to that for cells treated with control siRNA (graphs). The data shown are means ± SD from three independent experiments. A representative image of one well is also shown for all conditions. **(D)** Transfected cells were embedded in agar medium. One month later, the colonies formed were stained with MTT, photographed and counted. Colony counts are expressed as a percentage relative to the number of colonies obtained with cells treated with control siRNA (graph). Data are expressed as means ± SD, for triplicate measurements from two independent experiments. A representative image of one well is shown for all conditions. ^*^P<0.05, ^**^P<0.01, ^***^P<0.001 (i.e. a decrease with respect to the control siRN A).

Given the stronger expression of the LRP5 and LRP6 coreceptors in TNBC than in other breast cancer subtypes, we then investigated the effects of knocking down LRP5 or LRP6 expression on cell viability in HCC38 and MDA-MB-468 TNBC cells, which have high levels of both LRP5 and LRP6 [30]. We used two independent siRNAs to silence LRP5 and LRP6 expression in TNBC cells. Treatment with a siRNA against LRP6 decreased LRP6 protein levels (Figure 2A). The use of a siRNA against LRP5 decreased LRP5 protein levels, as expected, but also, to a lesser extent, those of the LRP6 protein (Figure 2A). It therefore seems possible that changes in LRP5 RNA levels could in turn modulate the expression of LRP6 at the protein level.

In both MDA-MB-468 and HCC38 cells, the depletion of LRP5 or LRP6 decreased cell viability (Figure 2B) and colony formation (Figure 2C). The two LRP5 siRNAs (#2 and #4) had similar impacts on cell viability, whereas one of the two LRP6 siRNAs (#8) had a stronger effect than the other (#7), possibly because it decreased LRP6 levels more strongly, as observed in MDA-MB-468 cells (Figure 2A). We also found that the depletion of these receptors decreased the viability of another TNBC cell line, MDA-MB-231 (data not shown), as previously reported for LRP6 invalidation [27]. We then investigated whether the depletion of LRP5 or LRP6 affected the tumorigenic properties of MDA-MB-468 cells *in vitro*, in a soft-agar assay in which the cells were allowed to grow in an anchorage-independent manner. We did not use HCC38 cells for these assays, because they do not form colonies in the conditions used. For both LRP5 and LRP6, silencing significantly decreased colony formation (Figure 2D).

Thus, both LRP5 and LRP6 are important for cell survival and have tumorigenic properties *in vitro*.

### The depletion of LRP5 or LRP6 induces apoptosis in breast cancer cells

**Figure 3:**
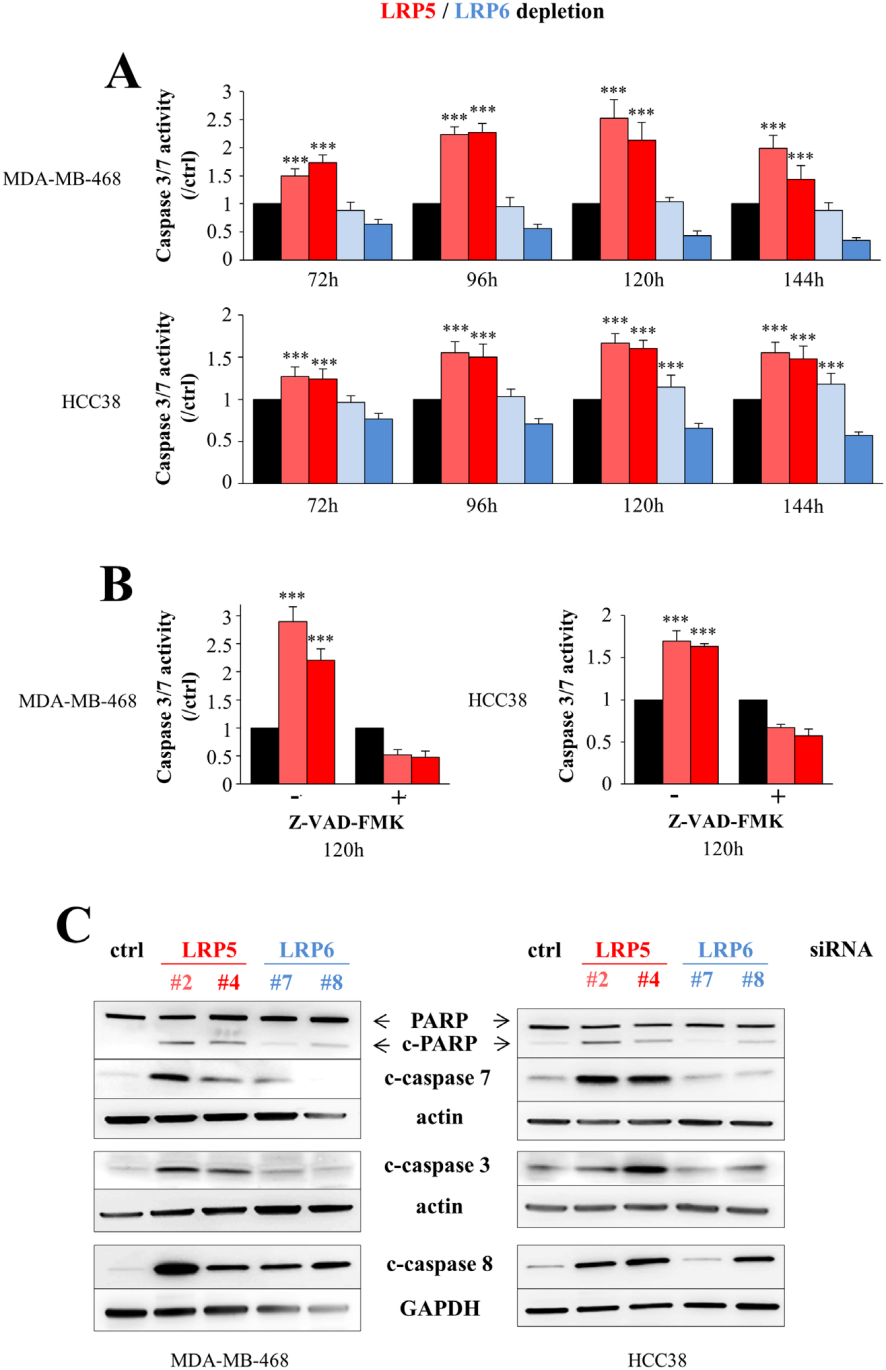
LRP5 depletion induces apoptosis more robustly than LRP6 depletion MDA-MB-468 and HCC38 cells were transfected with one of two different siRNAs against LRP5 (^#^2 and ^#^4, red) or LRP6 (^#^7 and ^#^8, blue) or with a control siRNA (black). **(A)** The activation of executioner caspases 3/7 was assessed 72 to 144 h after transfection, in a Caspase-Glo®3/7 luminescence assay. The results are presented as caspase3/7 activity normalized against the caspase activity in control siRNA-transfected cells. The data shown are the means ± SD for three independent experiments. In some conditions, caspase activity levels were lower than those in the control, because the results obtained in this assay depend in part on the number of cells. **(B)** The activation of caspases 3/7 in the presence or absence of the pan-caspase inhibitor Z-VAD-FMK was assessed 120 hours after the depletion of LRP5, as in Figure 3A. The data shown are means ± SD for 2 independent experiments performed in triplicate. **(C)** PARP cleavage (c-PARP) and the activation of caspases 3, 7 and 8 were analyzed by western blotting 96 hours after transfection with control (ctrl), LRP5 or LRP6 siRNAs. One representative experiment of the five performed, all of which gave similar results, is shown. Actin and GAPDH were used as loading controls. ^***^P<0.001 (i.e. an increase relative to control conditions).

We then investigated whether the decrease in cell viability observed following the depletion of LRP5 or LRP6 (Figure 2) resulted from programmed cell death, as we observed no defect in cell cycle progression (data not shown). Significant activation of caspases 3/7 was observed in MDA-MB-468 and HCC38 cells following the siRNA-mediated silencing of LRP5 (Figure 3A), and this activation was abolished by the presence of the pan-caspase inhibitor Z-VAD-FMK (Figure 3B). Western-blot analysis showed that LRP5 depletion led to the activation of caspases 3, 7 and 8, and to the cleavage of poly(ADP-ribose) polymerase (PARP), a substrate of caspases 3/7 (Figure 3C). The effect of LRP6 depletion on caspase 3/7 activity was not obvious in the caspase-Glo 3/7 luminescence assay (Figure 3A). However, this assay, in which cell number is crucial, is not suitable for detecting the low-level induction of apoptosis. By contrast, western-blot analysis indicated that LRP6 depletion induced the activation of caspases 7 and 8, albeit to a lesser extent than in LRP5-depleted cells (Figure 3C).

In conclusion, we show that LRP5 depletion induces apoptosis more strongly than LRP6 invalidation.

### LRP5 depletion reduces the expression of serine/threonine 40 (STK40)

We investigated the reasons for the stronger induction of apoptosis in cells depleted of LRP5, by using Affymetrix microarrays to analyze the transcriptome of HCC38 cells 24 h, 48 h and 72 h after the depletion of LRP5 or LRP6, to identify genes specifically regulated by LRP5. This analysis was performed in HCC38 cells, in which the activation of caspases 7 and 8 following LRP5 depletion with both siRNAs was stronger than in MDA-MB-468 cells (Figure 3C).

**Figure 4:**
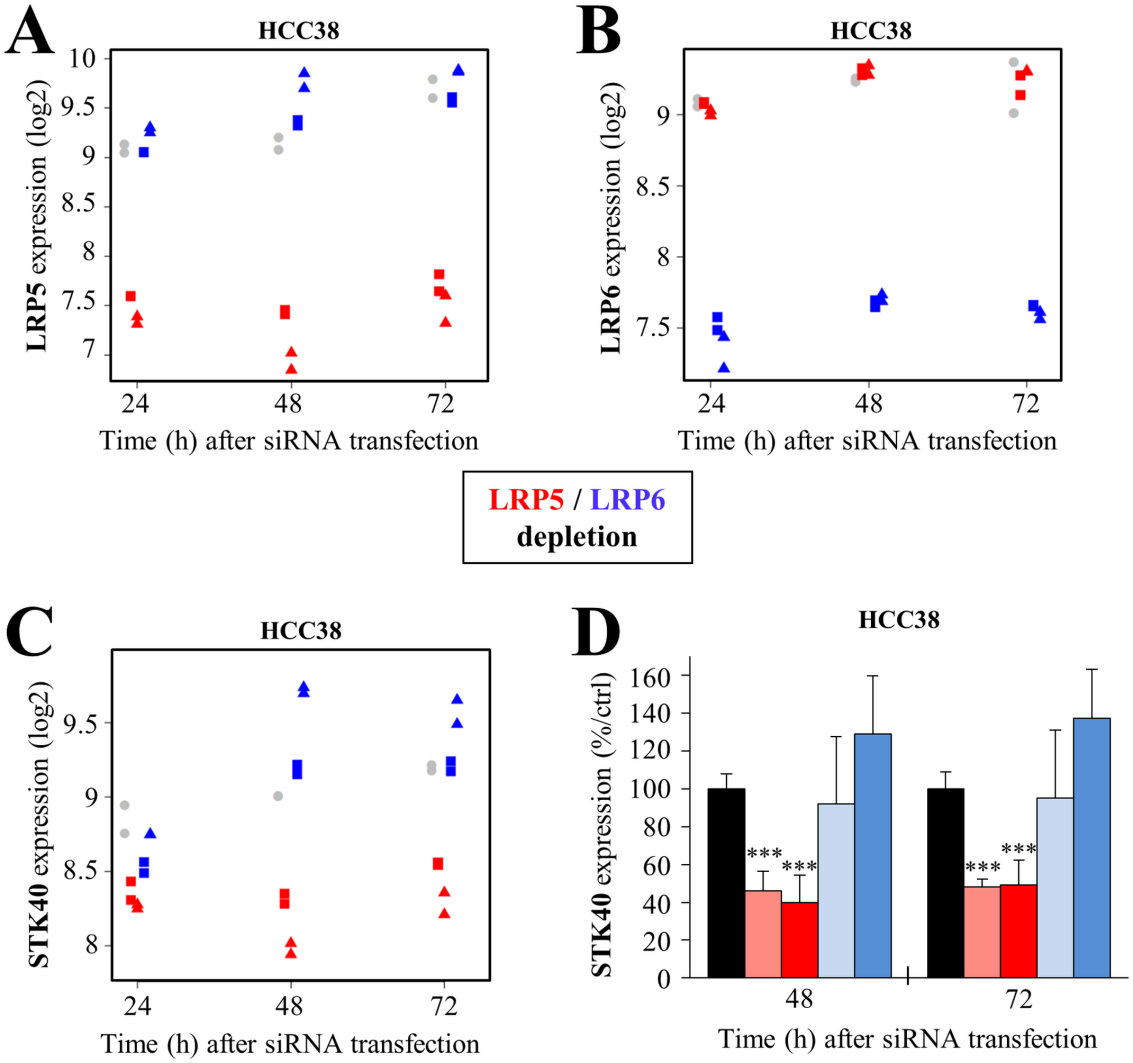
LRP5 regulates STK40 expression at the transcriptomic level **(A-C)** HCC38 cells were transfected with one of two different siRNAs against LRP5 (^#^2: red square; ^#^4, red triangle) or LRP6 (^#^7: blue square; ^#^8, blue triangle) or with a control siRNA (gray circle). This experiment was performed in duplicate. Expression profiling was performed with Gene 2.1 Affymetrix chips, for HCC38 cells 24 h, 48 h and 72 h after transfection. (A-B) The expression of LRP5 (A) or LRP6 (B) was first checked in LRP5- and LRP6-depleted cells, to validate the experiment. (C) Microarray analysis revealed that STK40 RNA levels were lower specifically in LRP5-depleted HCC38 cells. **(D)** We confirmed, by RT-qPCR, that STK40 RNA levels were specifically lower in HCC38 cells depleted of LRP5. The data shown are means ± SD from three independent experiments. ^*^P<0.05, ^**^P<0.01, ^***^P<0.001 (i.e. a decrease relative to the control siRN A).

We first validated the experiment by confirming the downregulation of LRP5 (Figure 4A) and LRP6 (Figure 4B) in cells treated with the corresponding siRNAs. The LRP5 and LRP6 siRNAs specifically depleted the cells of LRP5 and LRP6 RNA, respectively, demonstrating the specificity of the siRNAs (Figure 4A, 4B). We then identified genes that were deregulated in the same way by the two LRP5 siRNAs or by the two LRP6 siRNAs, with a fold-change ≥1.3 relative to control siRNA. We focused our analysis on the earliest time point (24 h), to avoid, as far as possible, genes displaying secondary deregulation due to the impairment of cell survival. Very few genes were found to be deregulated at 24 h (Supplementary Table 1). Some were specifically down- (STK40, ADAMTS15) or upregulated (ZNF367, HEATR1, INPP5A, ACVR1C, C5orf43) upon LRP5 depletion (Supplementary Table 1). Five genes were downregulated (LARP4, MDM2, CNTN5, FLRT3, HSDL1) and three were upregulated (IFFO2, CFDP1, FBN1) specifically after the depletion of LRP6 (Supplementary Table 1). No gene downregulated by the depletion of both LRP5 and LRP6 was identified, whereas four genes (DESI2, CSGALNACT2, RAB11FIP2, VPS4B) were upregulated 24 h after the depletion of both receptors (Supplementary Table 1).

The STK40 and ADAMTS15 mRNAs were the only transcripts specifically downregulated by LRP5 depletion at 24 h in HCC38 cells (Supplementary Table 1, Figure 4C). The proteins encoded by these transcripts may, therefore, be important for the LRP5-dependent effect on cell survival. We then focused on STK40, because this protein could potentially be targeted by a small-molecule approach. Microarray analysis revealed that STK40 levels were also lower at later time points (48 h, 72 h), and that the decrease relative to the control was even greater than at 24 h (Figure 4C). RT-qPCR confirmed that LRP5 depletion specifically decreased STK40 levels in HCC38 cells (Figure 4D).

Together, these results indicate that LRP5 depletion affects STK40 transcript levels.

### STK40 expression is altered in cancers, and is stronger in a subset of triple-negative breast cancers than in other breast cancer subtypes

STK40 belongs to the Ca^2+^/calmodulin-dependent protein kinase (CAMK) family. This protein is largely uncharacterized and its function remains unknown. STK40 has never been studied in the context of cancer.

**Figure 5:**
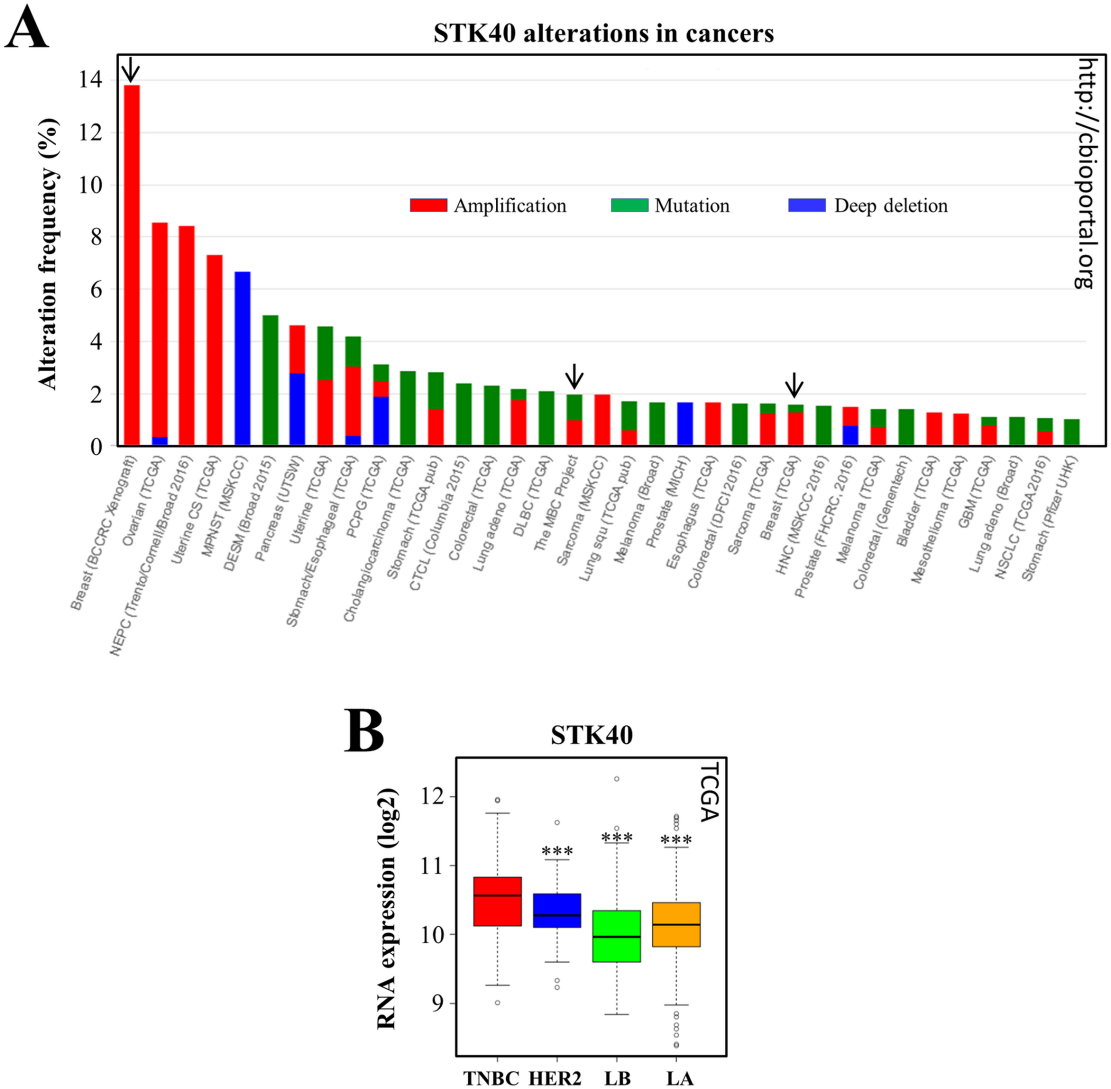
STK40 is amplified/mutated in various tumors and more strongly expressed in TNBC than in other subtypes of breast cancer **(A)** We queried the cBio Cancer Genomics Portal (http://cbioportal.org) [54, 55] to determine whether STK40 was altered in various types of cancer. The graph imported from cbioportal shows the cancer types in which STK40 alterations (cutoff ≥ 1%) have been identified (green: mutation; blue: deletion; red: amplification). The arrows indicate the breast cancer studies where STK40 alterations were found. CAN: DNA copy number alteration, BCCRC: breast cancer patient xenografts; NEPC: neuroendocrine prostate cancer; MPNST: malignant peripheral nerve sheath tumor; DESM: desmoplastic melanoma; PCPG: pheochromocytoma and paraganglioma; CTCL: cutaneous T-cell lymphoma; DLBC: lymphoid neoplasm diffuse large B-cell lymphoma; MBC: metastatic breast cancer; HNC: head and neck cancer; GBM: glioblastoma multiforme; NSCLC: non-small cell lung cancer. **(B)** We analyzed STK40 expression in the various breast tumor subtypes of the TCGA cohort [80]. The values obtained for the relative quantification of RNA were log-transformed and are shown as box plots. Outliers are shown within each population studied (open circles). ^***^P<0.001 (comparisons with TNBC).

We first investigated possible alterations to STK40 in cancers by interrogating the cBio Cancer Genomics Portal (http://cbioportal.org) [57, 58]. We found that some mutations, and, more rarely, deletions, had already been reported for the *STK40* gene (Figure 5A). Moreover, STK40 is overexpressed in various cancers, including ovarian and uterine carcinomas (Figure 5A). STK40 alterations were also identified in breast cancers (metastatic breast cancer project, TCGA cohort) (Figure 5A). Interestingly, the highest frequency of STK40 overexpression is that in breast cancer PDX (Figure 5A) and the reported amplifications were specifically observed in TNBC PDX models (http://cbioportal.org, [58]).

We then investigated STK40 expression in the different breast cancer subtypes. This analysis was not possible in our cohort, because STK40 was not retrieved in our analysis, possibly due to the poor quality of the STK40 probe set. We therefore looked at STK40 expression data for the publicly available TCGA cohort, which showed that STK40 was more strongly expressed in a subset of TNBCs than in other breast cancer subtypes (Figure 5B).

Overall, these analyses indicate that STK40 is highly expressed in various cancers, including breast cancers, with stronger expression in TNBCs than in other breast cancer subtypes.

### STK40 is a pro-survival protein kinase in breast cancer cells

**Figure 6:**
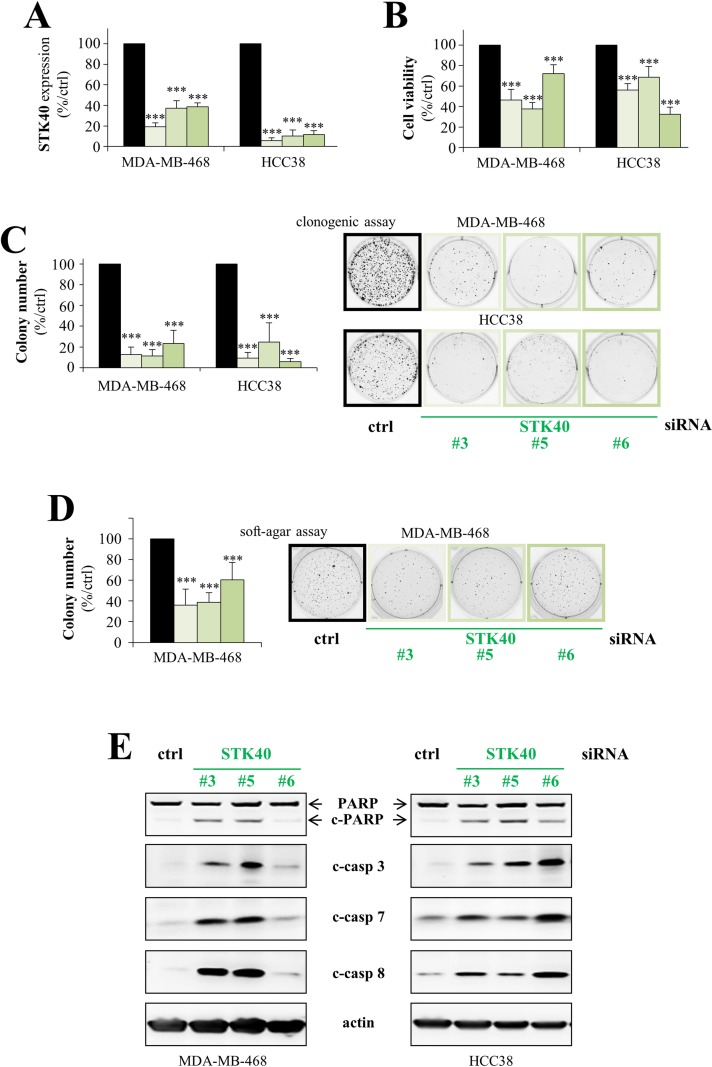
STK40 is a pro-survival protein kinase in breast cancer cells MDA-MB-468 and HCC38 cells were transfected with control siRNA (black) or with one of three different siRNAs against STK40 (green: ^#^3, ^#^5, ^#^6). **(A)** RT-qPCR analysis showing that transfection with these three STK40 siRNAs results in lower levels of STK40 RNA. The data shown are means ± SD from three independent experiments. **(B)** Cell viability was assessed in MTT (MDA-MB-468) or WST-1 (HCC38) assays, 144 hours after transfection. Results are presented as percent cell viability relative to cells treated with control siRNA (100%). The data shown are means ± SD from three independent experiments. **(C)** The cells were transfected and then cultured in six-well plates for 6-10 days, until colony formation. The number of colonies is presented as a percentage relative to that in cells treatment with the control siRNA (graphs). The data shown are means ± SD from three independent experiments. A representative image of one well is also shown for all conditions. The 2 images for ctrl siRNAs are the same than those shown in Figure 2C as the experiments were performed at the same time and therefore all the LRP5, LRP6 and STK40 siRNA shared the same control siRNAs. **(D)** Transfected MDA-MB-468 cells were embedded in agar medium. One month later, the colonies formed were stained with MTT, photographed and counted. The number of colonies is expressed as a percentage relative to that for cells treated with control siRNA (graph). The data are expressed as means ± SD from three independent experiments. A representative image of one well is shown for all conditions. **(E)** We analyzed the cleavage of PARP (c-PARP), caspase 3 (c-casp3), caspase 7 (c-casp7) and caspase 8 (c-casp8) by western blotting, 96 hours after transfection. One representative experiment of the three performed, all of which gave similar results, is shown. Actin was used as a loading control. ^*^P<0.05, ^**^P<0.01, ^***^P<0.001 (i.e. a decrease relative to the control siRN A).

As LRP5 depletion downregulates STK40 and induces apoptosis, we hypothesized that the lower levels of STK40 might inhibit proliferation and lead to the induction of apoptosis following LRP5 knockdown. Three different STK40 siRNAs were used and, as expected, they all decreased STK40 RNA levels following the transfection of MDA-MB-468 and HCC38 cells (Figure 6A). STK40 depletion decreased cell viability (Figure 6B) and had an even stronger inhibitory effect on colony formation (Figure 6C). STK40 depletion also inhibited colony formation in a soft-agar assay in an anchorage-independent manner (Figure 6D). Like LRP5 depletion (Figure 3), STK40 depletion induced apoptosis, with the activation of caspases 3, 7 and 8, and the cleavage of PARP (Figure 6E).

Overall, these results indicate that the little-studied protein kinase STK40 is crucial for breast cancer cell survival.

### Depletion of LRP5, LRP6 or STK40 slows tumor growth

**Figure 7:**
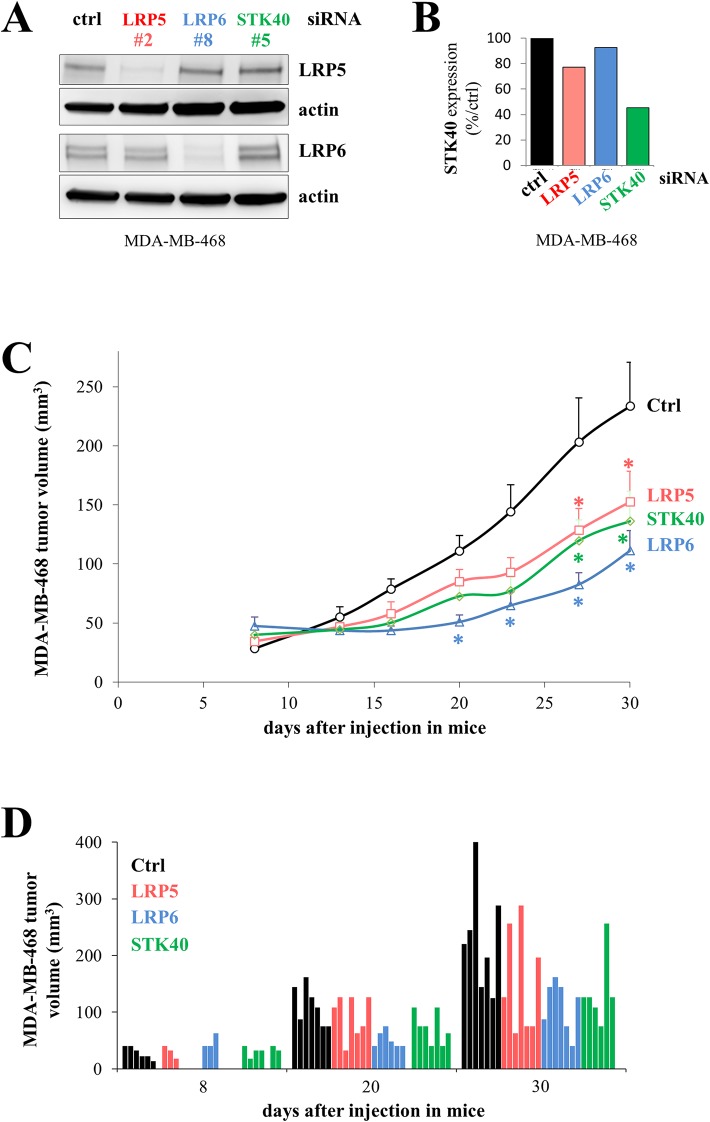
The depletion of LRP5, LRP6 or STK40 delays tumor growth MDA-MB-468 cells were transfected with control (ctrl, black), LRP5 (^#^2, red), LRP6 (^#^8, blue) or STK40 (^#^5, green) siRNAs. **(A)** The levels of LRP5 or LRP6 protein were evaluated by western blotting 24 hours after transfection. Actin was used as a loading control. **(B)** STK40 RNA levels were assessed by RT-qPCR analysis 24 hours after transfection with siRNA **(C)** Twenty-four hours after transfection, 4×10^6^ MDA-MB-468 cells were injected subcutaneously into Swiss *nude* mice (7 animals/group). Tumor growth was evaluated twice weekly for one month. The data shown are means + SD. The differences between the control siRNA and the other siRNAs (LRP5, LRP6 or STK40) were tested at each time point using an anova model on the raw data and on the square-rooted data, and adjusted for multiple testing using the Benjamini Hochberg correction (Supplementary Figure 1). Differences were considered significant if the adjusted *P* value was below 0.05 with both raw and squared-rooted data (indicated by ^*^: LRP5 and STK40: days 27 and 30; LRP6: days 20, 23, 27, 30). **(D)** Tumor volume is indicated for each animal at three time points (8, 20 and 30 days) following the injection of the transfected cells.

We investigated the effect of depleting LRP5, LRP6 or STK40 with siRNA in MDA-MB-468 cells on the tumorigenic potential of these cells following their injection into mice. This *in vivo* experiment was not performed with HCC38 cells, as these cells form very small tumors with slow growth when injected into female immunodeficient mice. We first checked that LRP5, LRP6 and STK40 levels were indeed reduced by transfection with the LRP5, LRP6 and STK40 siRNAs, respectively (Figure 7A, 7B). LRP5 and LRP6 levels were assessed by western blotting (Figure 7A), and STK40 expression was assessed by RT-qPCR, because the available anti-STK40 antibodies were not suitable for use in immunoblot analysis (Figure 7B). Depletions of LRP5, LRP6 or STK40 delayed tumor growth to similar extents in a statistically different manner (Figure 7C, Supplementary Figure 1). We analyzed seven mice per group, and, as expected for *in vivo* experiments, we observed some variability within each group (Figure 7D, Supplementary Figure 1).

These results support the previously expressed view that LRP6 could be used as a target in the treatment of TNBC [27], and suggest that LRP5 and STK40 are also potential, as yet unexplored treatment targets.

## DISCUSSION

The overexpression of some transmembrane receptors may underlie the activation of the Wnt/β-catenin signaling pathway in TNBC [35, 36]. In particular, LRP6 has been shown to be essential for tumorigenicity *in vitro* and *in vivo*, resulting in the proposal of its use as a treatment target for TNBC [26, 27]. As LRP5 and LRP6 are coreceptors, we analyzed the expression of these two receptors in breast cancers and the effects of their depletion on the survival of breast cancer cells.

We found that both LRP5 and LRP6 were more strongly expressed in TNBCs than in any other breast tumor subtype, consistent with published findings for LRP6 [26, 27]. To our knowledge, this is the first time that high levels of LRP5 have been reported in TNBC. A truncated form of LRP5 has been found in more than 80% of breast tumors [59]. However, like Chin and coworkers [49], and despite extensive investigations, we were unable to detect this truncated form in tumors or cell lines (unpublished data).

The knockdown of LRP5 or LRP6 expression in TNBC cell lines led to decreases colony formation and in the number of viable cells. We investigated whether the silencing of both LRP5 and LRP6 impaired cell viability to a greater extent than the depletion of LRP5 or LRP6 alone, but this was not found to be the case (data not shown). The knockdown of LRP5 or LRP6 reduced colony formation by TNBC cells in an anchorage-independent manner, suggesting that these coreceptors have tumorigenic properties. This hypothesis was confirmed *in vivo* in a xenograft model in which the depletion of LRP5 or LRP6 delayed tumor growth. These results are consistent with previous studies showing the coreceptors to be tumorigenic. Indeed, mice lacking LRP5 form Wnt1-induced mammary tumors much later than wild-type mice [60]. LRP6 overexpression in the mouse mammary gland induces mammary hyperplasia, whereas LRP6 downregulation inhibits breast cancer tumorigenesis [27, 38]. Moreover, Wnt1-induced tumors form later in *LRP6^+/-^* mice than in *LRP6^+/+^* mice [26]. Overall, these data indicate that both LRP5 and LRP6 are potential oncogenic proteins and, consequently, candidate treatment targets, as already suggested for LRP6 [27]. Mesoderm development (Mesd), which binds directly to mature LRP5 and LRP6 on the cell surface and inhibits Wnt3a-induced Wnt signaling [61], decreases the growth of breast cancer tumors *in vivo* [27]. However, Mesd cannot distinguish LRP5 from LRP6, and cannot be used to block LRP5 or LRP6 specifically. Antibodies inhibiting the interaction of LRP6 with Wnt1 or Wnt3a decrease breast tumor growth in Wnt1- and Wnt3a-driven xenografts, respectively [40, 41]. A bispecific antibody against LRP6, blocking its stimulation in the presence of Wnt and R-spondin ligands, has been shown to delay tumor growth *in vivo* in a patient-derived xenograft model of colorectal cancer [42]. These results indicate that the specific targeting of LRP6 with antibodies is a promising approach to limiting tumor growth [40–42]. A similar approach could be developed for LRP5, by generating antibodies antagonizing the function of this protein. Indeed, it may be more interesting to target LRP5 rather than LRP6 in TNBC, because LRP5 depletion had a more marked effect on cell death. Moreover, the potential side effects associated with treatments targeting these receptors are likely to be more severe for LRP6, given its crucial role in Wnt signaling for the maintenance of adult tissues. The potential adverse effects on bone of targeting LRP5 [62] could be managed by appropriate treatments, such as sclerostin-neutralizing antibodies [63].

The LRP5 and LRP6 coreceptors are homologous. They may have similar functions, but they may also be involved in different pathways. Indeed, LRP6-null mice die at birth [64], indicating that LRP5 cannot compensate for the loss of LRP6 during embryogenesis. By contrast, LRP5-deficient mice are viable, but develop Wnt1-induced mammary tumors much later than control mice, suggesting that LRP6 cannot compensate for LRP5 loss [60]. Experiments with LRP5/6 chimeric proteins have revealed that the cytoplasmic regions play a major role in the differences in signaling activity between the coreceptors [48]. A recent study showed that the depletion of LRP5 impaired the proliferation of mammary epithelial cells, whereas the depletion of LRP6 had no such effect [49]. The authors found that LRP5 controlled cell growth, not through the Wnt pathway, but through its function in glucose uptake [37]. However, they did not evaluate apoptosis in cells depleted of LRP5 or LRP6 [49]. They found that LRP5 depletion increased the activity of p38, inhibited the mTOR pathway, and impaired synthesis of the GLUT1 and GLUT8 transcripts [49]. LRP5 depletion did not affect the expression of these two glucose transporters in our transcriptomic analysis (data not shown), but we analyzed different cell lines. In future work, we will investigate whether LRP5 depletion leads to p38 activation and mTOR pathway inhibition in TNBC cells. The effects of LRP6 depletion on cell migration and invasion have recently been reported to be stronger than those on proliferation/viability [39].

We found that STK40 mRNA levels were decreased by the depletion of LRP5, but not by that of LRP6. This finding again highlights differences between the coreceptors. As LRP5 is a transmembrane receptor, the regulation of STK40 expression probably results from an indirect secondary effect, rather than a specific role of LRP5-mediated transactivation. However, the translocation of a soluble intracellular domain of LRP6 (LRP6-ICD) into the nucleus has been reported to regulate transcriptional activity [65]. There may also be a soluble form of LRP5-ICD involved in the regulation of transcription, but this remains to be demonstrated. Chromatin immunoprecipitation with antibodies against LRP5 may make it possible to determine whether LRP5 regulates STK40 expression directly. We have not yet investigated whether LRP5 overexpression induces STK40 expression. Clearly, additional experiments are required for an understanding of the molecular mechanisms underlying the regulation of STK40 expression by LRP5.

In 2003, STK40 was identified as a new SINK-homologous serine/threonine protein kinase (SHIK) impairing TNF-induced NF-κB activation [66]. Its precise function remains to be determined, but STK40 seems to play a role in cell differentiation. Indeed, STK40 induces embryonic stem cell differentiation [67], and is essential for mouse embryonic development [68]. STK40 knockout mice display respiratory failure, possibly due to inadequate epithelial cell differentiation [69]. STK40 is involved in the differentiation of skeletal muscle [70] and adipocytes [71]. STK40 interacts with RCN2, leading to activation of the MAPK/ERK pathway [67, 69]. Durzynska and coworkers recently reported an interaction between STK40 and the E3 ubiquitin ligase COP1 [72]. STK40 expression is regulated by miR-31 [73–76], and miR-31 levels were recently reported to be lower in tumors than in the adjacent tissue in TNBC patients [77]. The potential link between STK40 and miR-31 levels therefore remains to be studied in TNBC. STK40 has not yet been studied in the field of cancer, but our findings indicate that it is overexpressed, not only in TNBC, but also in other types of cancer, implying a possible role in tumor induction and/or progression. STK40 depletion inhibited cell proliferation and decreased the ability of TNBC cells to form colonies. STK40 depletion impaired colony formation in an anchorage-independent manner in a soft-agar assay, a hallmark of carcinogenesis. STK40 depletion also induced apoptosis. Cells lacking STK40 behaved similarly to cells depleted of LRP5. These results suggest that LRP5 depletion may exert its effect on cell viability, at least partly, by downregulating STK40. Further studies are required to test this hypothesis, by determining whether STK40 overexpression in cells lacking LRP5 can rescue cell survival, for example. However, we believe that the molecular mechanisms of the LRP5 survival pathway are more complex, and do not act solely through STK40. It also remains unclear whether STK40 itself regulates the Wnt pathway.

Overall, our results identify STK40 as a potential treatment target in TNBC. It may be possible to invalidate STK40 by inhibiting its kinase activity through a small-molecule approach. The crystal structure of a partial sequence of STK40 has recently been determined; it showed that STK40 was a pseudokinase with the potential to act as a scaffold protein [72]. However, the authors did not rule out the possibility that full-length STK40 functioned as an active kinase [72]. The possible requirement of the putative kinase activity of STK40 for cell survival could be assessed by evaluating the extent to which the overexpression of a form of STK40 mutated in the catalytic loop, as opposed to wild-type STK40, rescues cell survival in cells depleted of STK40. Alternatively, if STK40 is, indeed, a pseudokinase that acts as a scaffold protein, it may be possible to invalidate it by targeting its binding to protein partners. In this case, it would be essential to identify the proteins binding to STK40.

In summary, we found that both LRP6 and LRP5 were candidate treatment targets for TNBC. LRP5 depletion induced apoptosis more effectively than LRP6 knockdown, suggesting that it may be more efficient to target LRP5. However, the depletion of LRP5 had a similar effect to that of LRP6 on tumor growth in a xenograft model. LRP5 and LRP6 are coreceptors, but it has been suggested that they may also have specific functions. We report here that STK40 levels decrease in a specific manner following the depletion of LRP5, but not that of LRP6. We found that STK40 was more strongly expressed in TNBC than in other breast cancers, and that this molecule was overexpressed in various other types of cancer. Its invalidation induced apoptosis, impaired proliferation and slowed tumor growth. Overall, our results identify STK40, a little-characterized protein, as a potential new treatment target in breast cancer.

## MATERIALS AND METHODS

### Human breast cancer sample cohorts

Our cohort, composed of 35 luminal A, 40 luminal B, 46 TNBC, and 33 HER2/ER^-^ tumor samples has been described elsewhere [30, 46, 47, 78, 79]. Experiments were performed in accordance with Bioethics Law No. 2004-800 and the Ethics Charter of the French National Cancer Institute (INCa), with approval from the ethics committee of our institution. Informed consent was not required; women were informed of the use of their tissues for research purposes and none declared their opposition to this use.

The TCGA breast invasive carcinoma (TCGA-BRCA) cohort is publicly available [80]. The RNA-SeqV2 Level 3 data (Jan 2015) were downloaded from the TCGA Research Network (http://cancergenome.nih.gov/) and integrated into a platform in knowledge data integration (KDI) at Institut Curie (https://bioinfo-portal.curie.fr). Subtype classification was based on immunohistochemical status for the estrogen receptor (ER), progesterone receptor (PR) and HER2, as follows. TNBC: ER-, PR- and HER2-negative (*n*=157); HER2^+^/ER^-^: ER- and PR-negative, HER2-positive (*n*=41); luminal B: ER- and/or PR-positive, HER2-positive (n=153); luminal A: ER- and/or PR-positive, HER2-negative (*n*=663).

### DNA microarray analysis (our cohort)

*LRP5* and *LRP6* DNA copy numbers were determined for the different tumor subtypes: TNBC (*n*=46), HER2^+^/ER^-^ (*n*=33), luminal A (*n*=35) and luminal B (*n*=39). DNA was extracted from frozen tumor samples by a standard phenol/chloroform-based procedure. Genomic DNA (500 ng) was processed for hybridization with Affymetrix SNP6.0 arrays in accordance with the manufacturer's recommendations. The collected data were then processed as described elsewhere [46, 47]. We defined the thresholds for gain and loss as the 0.999 and 0.001 quantiles of the distribution of smoothed probe signals for healthy breast tissue samples.

### RNA microarray analysis (our cohort)

We assessed LRP5 or LRP6 mRNA levels in the different tumor subtypes: TNBC (*n*=41), HER2^+^/ER^-^ (*n*=30), luminal A (*n*=29) and luminal B (*n*=30). Total RNA was extracted from frozen tumor samples with the RNeasy Mini Kit (Qiagen, Courtaboeuf, France) and was then processed with an RNA clean-up kit (Qiagen). The quality of the RNA was checked, and samples were then hybridized with U133 Plus 2.0 Affymetrix chips and processed in accordance with the manufacturer's instructions. The data were analyzed as described elsewhere [46, 47].

### Reverse-phase protein array (our cohort)

LRP6 protein levels were assessed in the different tumor subtypes: TNBC (*n*=42), HER2^+^/ER^-^ (*n*=28), luminal A (*n*=24) and luminal B tumors (*n*=37). The reverse-phase protein array (RPPA) is a miniaturized dot-blot technology based on the robotic printing of a large number of different cell/tissue lysates onto nitrocellulose bound to histology slides. It involves the printing of very small quantities of protein (about 1 ng per spot), and is convenient for the analysis of minimal quantities of biopsy material. The proteins of interest are detected with highly specific antibodies. Total protein was extracted from frozen tumor samples and processed according to a protocol described elsewhere [46, 47].

### RNA microarray analysis of cells depleted of LRP5 or LRP6

HCC38 cells were transfected with control, LRP5 (LRP5#2 or LRP5#4) or LRP6 (LRP6#7 or LRP6#8) siRNAs for 24, 48 or 72 hours. The experiment was performed in duplicate. Total RNA was extracted with the RNeasy Mini Kit from Qiagen (Courtaboeuf, France), according to the manufacturer's instructions. The quality and quantity of RNA obtained was assessed, and samples were hybridized with Affymetrix Gene 2.1 chips. Samples were processed as described on the company's website. The data were analyzed with brainarray hugene21sthsentrezg version 16 software [81]. The data were first log_2_-transformed and normalized by robust multiarray averaging (RMA) [82]. At each time point, the significance of differences between LRP5 or LRP6 siRNA-treated cells and control siRNA-treated cells was determined with LIMMA [83] and Benjamini & Hochberg correction for multiple testing [84]. Our analysis focused on genes displaying significant differential expression (*P* < 0.05) with a fold change > 1.3, or < 1.3 with both LRP5 siRNAs or both LRP6 siRNAs relative to control conditions, at the earliest time point considered (24 h; Supplementary Table 1). Transcriptomic data for LRP5- and LRP6-depleted HCC38 cells are available from Gene Expression Omnibus (GEO) (accession number: GSE109004).

### Cell culture

The HCC38 and MDA-MB-468 TNBC cell lines were purchased from the American Type Culture Collection (LGC Standards, Molsheim, France). MDA-MB-468 cells were maintained in RPMI-1640 containing Glutamax (Invitrogen) and supplemented with 10% FBS. HCC38 cells were cultured in RPMI-1640 containing Glutamax and supplemented with 10% FBS, 1.5 g/L sodium bicarbonate, 10 mM Hepes (Invitrogen) and 1 mM sodium pyruvate. Antibiotics were added to all media (100 U/mL penicillin and 100 μg/mL streptomycin). Cells were cultured at 37°C in a damp incubator, under an atmosphere containing 5% CO_2_.

### Transfection with small interfering RNAs (siRNAs)

Cells were transiently transfected with 20 nM siRNA (Qiagen, Courtaboeuf, France) in the presence of Lipofectamine™ RNAiMax reagent (Invitrogen), as described elsewhere [47, 85].

The siRNAs used were:

Allstars negative control (ref SI03650318)

LRP5#2 (SI00036239), target sequence: 5’-CCCGTTCGGTCTGACGCAGTA-3’.

LRP5#4 (SI00036253), target sequence: 5’-CTGGATGGGCAAGAACCTCTA-3’.

LRP6#7 (SI02628353), target sequence: 5’-CTGGATGGTTCTGACCGTGTA-3’.

LRP6#8 (SI03072188), target sequence: 5’-CAGGTGCTAACCGGATAGTAT-3’.

STK40#3 (SI04379648), target sequence: 5’-CGCCCGGAGCTGGGTACCCAA-3’.

STK40#5 (SI00287910), target sequence: 5’-CAGCGCTACCTGCGGAAATAA-3’.

STK40#6 (SI00287917), target sequence: 5’-CCGGATGGTTAAGAAGATGAA-3’.

### SDS-PAGE and western blotting

Cells were lysed in Laemmli buffer containing 50 mM Tris (pH 6.8), 2% sodium dodecyl sulfate (SDS), 5% glycerol, 2 mM 1,4-dithio-DL-threitol, 2.5 mM ethylenediaminetetraacetic acid, 2.5 mM ethylene glycol tetraacetic acid, 2 mM sodium orthovanadate, 10 mM sodium fluoride and a cocktail of protease (Roche) and phosphatase (Pierce, Perbio, Brebières, France) inhibitors. The protein concentration in each sample was determined with the reducing agent-compatible version of the BCA Protein Assay kit (Pierce). Equal amounts of total protein were fractionated by SDS-PAGE under reducing conditions, and blotted onto PVDF membranes (Bio-Rad, Marnes-la-Coquette, France). The membranes were blocked with 5% BSA or 10% skimmed milk in TBS containing 0.1% Tween 20 (TBS-T), and hybridized with the primary antibody of interest overnight at 4°C. Membranes were washed in TBS-T and then hybridized with the secondary antibody for one hour at room temperature. Antibodies were diluted in TBS-T containing 5% BSA or 10% skimmed milk. The membranes were washed with TBS-T, and immune complexes were detected by enhanced chemiluminescence (Amersham, GE Healthcare, Orsay, France). Actin and GAPDH were used as loading controls.

### Compounds, antibodies

The primary antibodies used for RPPA and/or western blotting were rabbit anti-LRP5, rabbit anti-LRP6, mouse anti-PARP, rabbit anti-caspase 3, rabbit anti-caspase 7, rabbit anti-cleaved caspase 8, rabbit anti-GAPDH (Cell Signaling Technology, Ozyme, Saint Quentin Yveline, France) and mouse anti-actin (Sigma) antibodies. The secondary antibodies used for RPPA and/or western blotting were horseradish peroxidase-conjugated anti-mouse IgG and anti-rabbit IgG (Jackson ImmunoResearch Laboratories, Interchim, Montluçon, France).

The broad-spectrum caspase inhibitor Z-VAD-FMK (R&D Systems) was dissolved in DMSO and used at a final concentration of 30 μM.

### Quantitative real-time reverse transcription-polymerase chain reaction (RT-qPCR)

Total RNA was extracted with the RNeasy Mini Kit (Qiagen). Samples were subjected to RT-qPCR according to the kit manufacturer's instructions (KapaBiosystems).

### Cell proliferation assays

WST-1 (Roche, Meylan, France) was added to HCC38 cells, according to the manufacturer's instructions. After incubation for one to four hours at 37°C, we measured absorbance at 440 nm (Infinite 200®, Tecan, Lyon, France). MTT (5 mg/mL in PBS, Sigma) was added to MDA-MB-468 cells according to the manufacturer's recommendations. After four hours of incubation at 37°C, a solution of 10% SDS in 10 mM HCl was added. The mixture was incubated overnight at 37°C, and absorbance was then measured at 540 nm. Both assays are based on the cleavage of tetrazolium salts to formazan by mitochondrial succinate dehydrogenases (present in metabolically active cells).

### Clonogenic assays

Cells transfected with siRNA were used to seed 2 ml of growth medium per well, in six-well plates. Cells were incubated at 37°C for 6-10 days, until colonies formed. Colonies were fixed and stained by incubation with 500 μl of Coomassie brilliant blue solution for 20 minutes. Colonies were photographed with a LAS-3000 Luminescent Image Analyzer (Fuji, FSVT) and quantified with ImageJ 1.43u software (NIH).

### Caspase assay

Caspase activity was determined in Caspase-Glo^®^3/7 luminescence assays (Promega, Charbonnières-les-Bains, France), according to the kit manufacturer's instructions. Luminescence was recorded on an Infinite 200^®^ plate reader. The results of this assay are dependent on the number of cells.

### Soft-agar assay

The impact of LRP5, LRP6 or STK40 downregulation on tumorigenesis was evaluated *in vitro*, by analyzing cell growth in an anchorage-independent manner. We added a 1 ml bottom layer of medium containing 0.5 % agar (equal volumes of 1% agar and 2× culture medium) to six-well plates. MDA-MB-468 cells were transfected with siRNA, treated with trypsin 24 h later, resuspended in 0.35% agar medium, and plated at a density of 5000 cells/well, as a top layer. Cells were incubated for 4 weeks at 37°C, and the colonies were stained in an MTT assay. Plates were photographed with a Fujifilm LAS-3000 Imager, and clones were counted with Image J software. HCC38 cells did not form colonies under these conditions (data not shown).

### Mouse and measurement of tumor growth

Five- to six-week-old female Swiss *nude* mice were purchased from Charles River (Les Arbresles, France) and maintained in specific pathogen-free conditions. The care and use of mice complied with the internationally recognized principles of replacement, reduction and refinement, and with UKCCCR guidelines, in particular [86]. The protocol was validated by the local ethics committee. MDA-MB-468 cells (4×10^6^) were injected subcutaneously into the mice 24 hours after LRP5, LRP6 or STK40 depletion (7 mice/group). Tumor growth was evaluated by measuring two perpendicular tumor diameters with a caliper, twice weekly. Tumor volume (V) was calculated as follows: V=a×b^2^/2; a and b being the largest and smallest perpendicular tumor diameters, respectively. We minimized the number of animals used, by investigating the effects on tumor growth of only one siRNA each for LRP5, LRP6 STK40 targeting. LRP5#2, LRP6 #8 and STK40#5 were chosen for this experiment, as they gave the highest levels of caspase activity *in vitro*.

### Statistical analyses

For the human samples, differences between groups were assessed with Fisher's exact test (DNA microarray) or with an anova model (RNA, RPPA microarrays) and considered significant if the *P* value was below 0.05.

For cellular assays, *P* values were calculated using the Student *t* test and considered significant if below 0.05.

For the *in vivo* experiment, we considered an anova model with 28 groups (7 time points, 4 different siRNA) and tested the difference between the control siRNA and the other siRNAs (LRP5, LRP6 or STK40) at each time point (making a total of 21 tests). To take into account that variances were higher for larger measurements, we also ran the anova model on the square-rooted data. We adjusted for multiple testing using the Benjamini Hochberg correction. Differences were considered significant if the adjusted *P* value was below 0.05 for both the raw-data model and the square-rooted data model.

## SUPPLEMENTARY MATERIALS FIGURE AND TABLE





## Abbreviations

APC: adenomatous polyposis coli
ER: estrogen receptor
FZD6/7: frizzled receptor 6/7
HER2: human epidermal growth factor receptor 2
LRP5/6: low-density lipoprotein receptor-related protein 5/6
PARP: poly(ADP-ribose) polymerase
RT-qPCR: quantitative real-time reverse transcription-polymerase chain reaction
RPPA: reverse phase protein array
SD: standard deviation
siRNA: small interfering RNA
STK40: serine threonine kinase 40
TNBC: triple-negative breast cancer
