# Supplementary material for: LRP5 regulates the expression of STK40, a new potential target in triple-negative breast cancers
Source: Oncotarget. 2018 Apr 27;9(32):22586–604. doi: 10.18632/oncotarget.25187 (PMC5978250; doi:10.18632/oncotarget.25187)
Supplement: Supplementary file 1 [file oncotarget-09-22586-s001.pdf]

LRP5 regulates the expression of STK40, a new potential target in triple-negative breast cancers

SUPPLEMENTARY MATERIALS

	mice	Tumor volume (mm3)						
		Day 8	Day 13	Day 16	Day 20	Day 23	Day 27	Day 30
control siRNA	1	40	87.5	87.5	144	144	196	220.5
	2	32	75	126	162	269.5	416	416
	3	40	48	75	87.5	126	196	245
	4	0	0	62.5	75	126	162	288
	5	22.5	40	62.5	126	144	144	144
	6	13.5	32	62.5	75	75	112.5	125
	7	22.5	48	75	108	126	196	196
LRP5 siRNA	1	18	32	32	32	32	32	62.5
	2	0	32	40	62.5	62.5	62.5	75
	3	0	62.5	75	126	126	196	288
	4	40	40	62.5	108	75	108	126
	5	0	75	108	126	126	196	196
	6	32	75	75	126	126	171.5	256
	7	0	40	62.5	75	75	75	75
LRP6 siRNA	1	0	32	40	48	48	87.5	144
	2	0	0	0	0	40	87.5	126
	3	40	40	62.5	62.5	75	87.5	144
	4	0	0	40	40	62.5	62.5	75
	5	40	40	40	40	62.5	87.5	87.5
	6	0	0	40	40	40	40	40
	7	62.5	62.5	40	75	126	126	162
STK40 siRNA	1	40	40	62.5	108	126	126	126
	2	18	40	40	75	62.5	126	126
	3	32	32	62.5	62.5	108	NA	NA
	4	32	40	40	75	75	108	108
	5	32	32	32	40	62.5	75	75
	6	0	75	75	108	126	196	256
	7	40	40	40	40	72	87.5	126

	LRP5		LRP6		STK40	
	raw (adjusted *P* value)	square rooted (adjusted *P* value)	raw (adjusted *P* value)	square rooted (adjusted *P* value)	raw (adjusted *P* value)	square rooted (adjusted *P* value)
Day 8	0.782	0.152	0.889	0.301	0.889	0.831
Day 13	0.889	0.615	0.532	0.101	0.889	0.875
Day 16	0.747	0.584	0.168	**0.047**	0.385	0.236
Day 20	0.657	0.566	**0.015**	**0.006**	0.195	0.171
Day 23	0.052	0.096	**0.003**	**0.015**	0.054	0.110
Day 27	**0.003**	**0.032**	**0.000**	**0.003**	**0.003**	**0.047**
Day 30	**0.003**	**0.047**	**0.003**	**0.004**	**0.001**	**0.032**

Supplementary Figure 1: Tumor volumes and statistical analysis of the in vivo experiment (see Figure 7). Top table: tumor volume (mm^3) determined for each mice following injection with siRNA transfected MDA-MB-468 at different time points (day 8, day 13, day 16, day 20, day 23, day 27, day 30). NA: not applicable (the mouse n°3 of the STK40 group died during the experiment). **Bottom table:** Statistical analysis of the xenograft experiment. We considered an anova model with 28 groups (7 days of tumor measurement, 4 different siRNA) and tested the difference between the control siRNA and the other siRNAs (LRP5, LRP6 or STK40) at each time point (a total of 21 tests) (columns “raw”). To take into account that variances were higher for larger measurements, we also ran the anova model on the square-rooted data (columns “square rooted”). We adjusted for multiple testing using the Benjamini Hochberg correction. Differences were considered significant if the adjusted *P* value was below 0.05 (indicated in bold and red).

Supplementary Table 1: Analysis of gene expression in HCC38 cells depleted of LRP5 and LRP6. This table lists the genes specifically up- or downregulated by LRP5 or LRP6 depletion, and the genes displaying a change in expression following the depletion of both receptors 24 h after transfection with siRNA (fold change > 1.3 relative to the control siRNA). STK40 was one of two genes specifically downregulated by LRP5 depletion.

See Supplementary File 1
